# Clinically aligned artificial intelligence for glaucoma diagnosis: enhancing retinal nerve fibre layer interpretation from fundus images

**DOI:** 10.1038/s41433-026-04758-w

**Published:** 2026-07-23

**Authors:** Shang-Lin Chung, Jehn-Yu Huang, Yi-Ching Shao, Chien-Chia Su, Yun Hsia, Pai-Huei Peng, Pei-Yao Chang, Bo-I Kuo, Chien-Hung Li, Ming-Chi Kuo, Yi-Jin Huang, Wei-Hao Chang, Kai-Cheng Hsu, Chia-En Lien

**Affiliations:** 1 Acer Incorporated, New Taipei City, Taiwan, ROC; 2 Department of Ophthalmology, National Taiwan University Hospital, Taipei, Taiwan, ROC; 3 College of Medicine, National Taiwan University, Taipei, Taiwan, ROC; 4 Department of Ophthalmology, China Medical University Hospital, Taichung, Taiwan, ROC; 5 School of Medicine, College of Medicine, China Medical University, Taichung, Taiwan, ROC; 6 Graduate Institute of Biomedical Sciences, College of Medicine, China Medical University, Taichung, Taiwan, ROC; 7 Department of Ophthalmology, National Taiwan University Hospital, Hsinchu, Taiwan, ROC; 8 Department of Ophthalmology, Shin-Kong Wu Ho-Su Memorial Hospital, Taipei, Taiwan, ROC; 9 Far Eastern Memorial Hospital, New Taipei, Taiwan, ROC; 10 Acer Medical Inc., New Taipei City, Taiwan, ROC

**Keywords:** Eye abnormalities, Optic nerve diseases

## Abstract

**Background/objectives:**

This study aimed to develop an interpretable artificial intelligence (AI) screening system that replicates a specialist’s evaluation of fundus photographs. The system analyses three signs: visible retinal nerve fibre layer (RNFL) defects (as observed on fundus photography, not OCT-based measurement), vertical cup-to-disc ratio (VCDR) and rim-to-disc ratio (RDR).

**Subjects/methods:**

A total of 773 fundus images from the independent test cohort were annotated by three fellowship-trained glaucoma specialists, followed by repeated consensus meetings to improve annotation consistency. Two models were trained on the development cohort: an EfficientNet-B4 classifier for RNFL and a U-Net with an EfficientNet-B4 encoder for optic disc/cup (OD/OC) segmentation. During the testing phase, a referral was triggered whenever a feature was classified as abnormal, with the system explicitly reporting the specific sign that prompted the referral decision.

**Results:**

Among the 773 expert-annotated cases, 749 were included in the analysis after excluding low-quality or insufficient-information images (AI drop rate: 3.1%). The final independent test cohort comprised 268 referral and 481 non-referral cases, providing a basis for evaluation. The combined model achieved a sensitivity of 0.903 (95% confidence interval [CI], 0.868–0.938), specificity of 0.821 (95% CI, 0.787–0.855) and a positive predictive value (PPV) of 0.738 (95% CI, 0.690–0.785). The system captured complementary aspects of glaucomatous optic neuropathy that are often missed by single-feature approaches.

**Conclusions:**

Consensus-based annotation, combined with lesion-level modelling, enhances alignment with clinical reasoning. By providing an explicit referral rationale, the system fosters trust in AI-assisted glaucoma screening and facilitates adoption in clinical settings.

## Introduction

Glaucoma is a chronic, progressive optic neuropathy and a leading cause of irreversible blindness worldwide. The global prevalence is projected to increase with ageing populations, creating a substantial burden [1]. The disease typically progresses silently and many patients remain asymptomatic until advanced stages, when substantial visual field loss has already occurred. Consequently, more than half of individuals remain undiagnosed, particularly in regions without systematic screening [2]. Advanced glaucoma imposes substantial long-term healthcare costs. From a health economics perspective, effective early detection is essential to reduce the clinical and economic burden [3]. Despite this urgent need, widespread glaucoma screening has not been implemented in many healthcare systems, partly due to uncertainties regarding cost-effectiveness and the absence of a standardised, scalable screening modality [4].

The vertical cup-to-disc ratio (VCDR) has been widely used in epidemiological studies and clinical trials to define glaucomatous optic neuropathy (GON) [5–8]. Subsequent studies have emphasised the diagnostic value of the neuroretinal rim and the rim-to-disc ratio (RDR) [9–12], while peripapillary retinal nerve fibre layer (RNFL) defects are recognised as an even more sensitive marker [13–15]. However, reliable detection of RNFL defects on fundus photography requires the expertise of experienced specialists. Considering VCDR, RDR and RNFL defects together allows for a rigorous diagnosis of glaucoma.

Artificial intelligence (AI) has been widely applied to glaucoma detection, with key datasets such as the Online Retinal Fundus Image Database for Glaucoma Analysis (ORIGA) [16] and the Retinal Fundus Glaucoma Challenge (REFUGE) facilitating research and development [17]. While most studies primarily use optic disc and cup (OD/OC) features for classification [18–20], clinical diagnosis is inherently more comprehensive, incorporating multiple signs. To better emulate this process, some approaches employ referral-level labels from expert consensus [21], whereas others utilise optical coherence tomography (OCT) to derive RNFL references from fundus photographs [22, 23]. More recently, several studies have begun to combine RNFL with OD/OC features to reflect the integrated assessment performed by specialists [24]. However, a persistent limitation of these methods is their reliance on simple majority voting. Given that assessments of RNFL and OD/OC frequently vary between graders, agreement may decline to unacceptable levels without rigorous consistency training [25].

To address these challenges, this study aimed to develop a consensus-based annotation workflow incorporating structured, multi-round reviews. The model jointly learns from RNFL, VCDR and RDR, while integrating lesion-level features that reflect clinical reasoning and judgement. Occlusion-based explainability [26] was employed to visualise the image regions influencing model predictions and to assess whether the model focused on clinically relevant signs. This framework establishes an AI-driven screening system that combines widely accessible imaging with clinically relevant features, enabling earlier detection and alleviating the burden of glaucoma.

## Materials and methods

This study was conducted in accordance with the Declaration of Helsinki and received approval from two institutional review boards (201912017RIFA and 202404048DSA). The study design employed a development cohort for model development and an independent test cohort for temporally independent evaluation at a single centre.

### Cohorts, image acquisition and curation

**Fig. 1:**
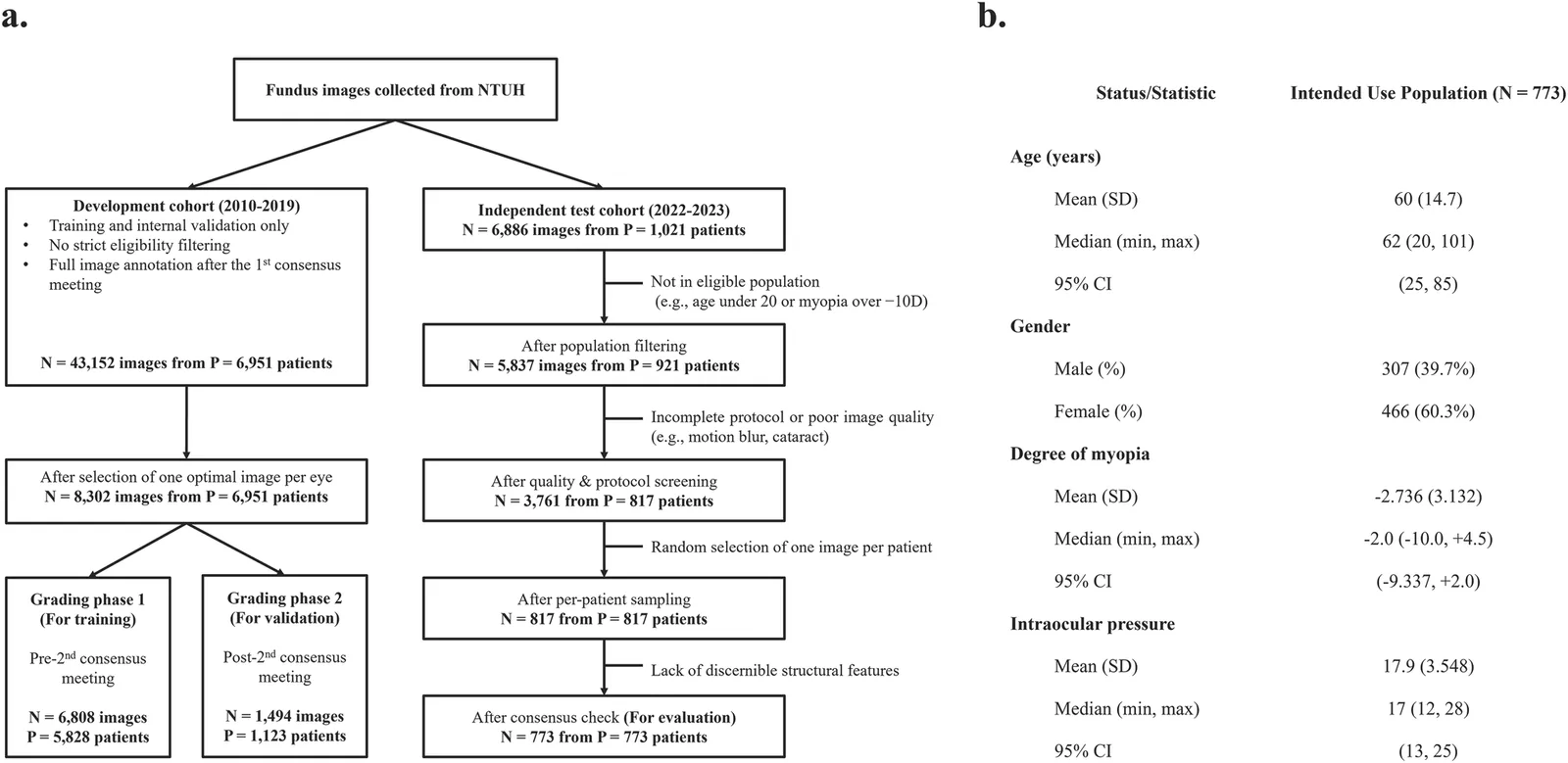
Study flow and patient characteristics of the dataset. **a** Cohort selection process for fundus photographs included in the independent test cohort. **b** Demographic and clinical characteristics of patients in the independent test cohort.

All fundus photographs were obtained at the Department of Ophthalmology, National Taiwan University Hospital, using a Canon CR-2 non-mydriatic camera. The camera provided a 45-degree field of view centred on the fovea, capturing the optic disc and macula for RNFL assessment. The study comprised two temporally distinct cohorts (Fig. 1a). The development cohort (2010 to 2019) included 43,152 images from 6951 patients and was used exclusively for model training and internal validation. Following quality screening, one optimal image per eye was randomly selected to prevent eye-level data leakage. The final development cohort for training and validation consisted of 6808 and 1494 images, respectively. The development cohort is shown in Fig. 1a as two grading phases (before and after the second consensus meeting), reflecting the evolution of grading criteria through consistency training.

The independent test cohort (2022 to 2023) initially comprised 6886 images from 1021 patients and was reserved exclusively for blinded consensus review, ground truth construction and performance evaluation. To derive the final independent test cohort, a stepwise exclusion process was implemented. Initially, 1049 images from patients younger than 20 years or with high myopia worse than -10 dioptres were excluded, leaving 5837 images from 921 patients. Subsequently, quality and protocol screening removed 2076 images with motion blur, media opacity, or incomplete examinations, resulting in 3761 images from 817 patients. To avoid patient-level leakage, one representative image per patient was randomly selected, yielding 817 images. All images were then subjected to a standardised consensus grading procedure by glaucoma specialists. Images in which the OD or RNFL could not be assessed were further excluded from the final independent test cohort. However, some retained images were deemed uninterpretable during expert annotation if one of the assessed signs could not be reliably evaluated. Consequently, the final independent test cohort comprised 773 images from 773 patients, including 276 GON and 497 non-GON cases. Demographic and clinical characteristics of the independent test cohort are summarised in Fig. 1b.

### Expert annotation and consensus

**Fig. 2:**
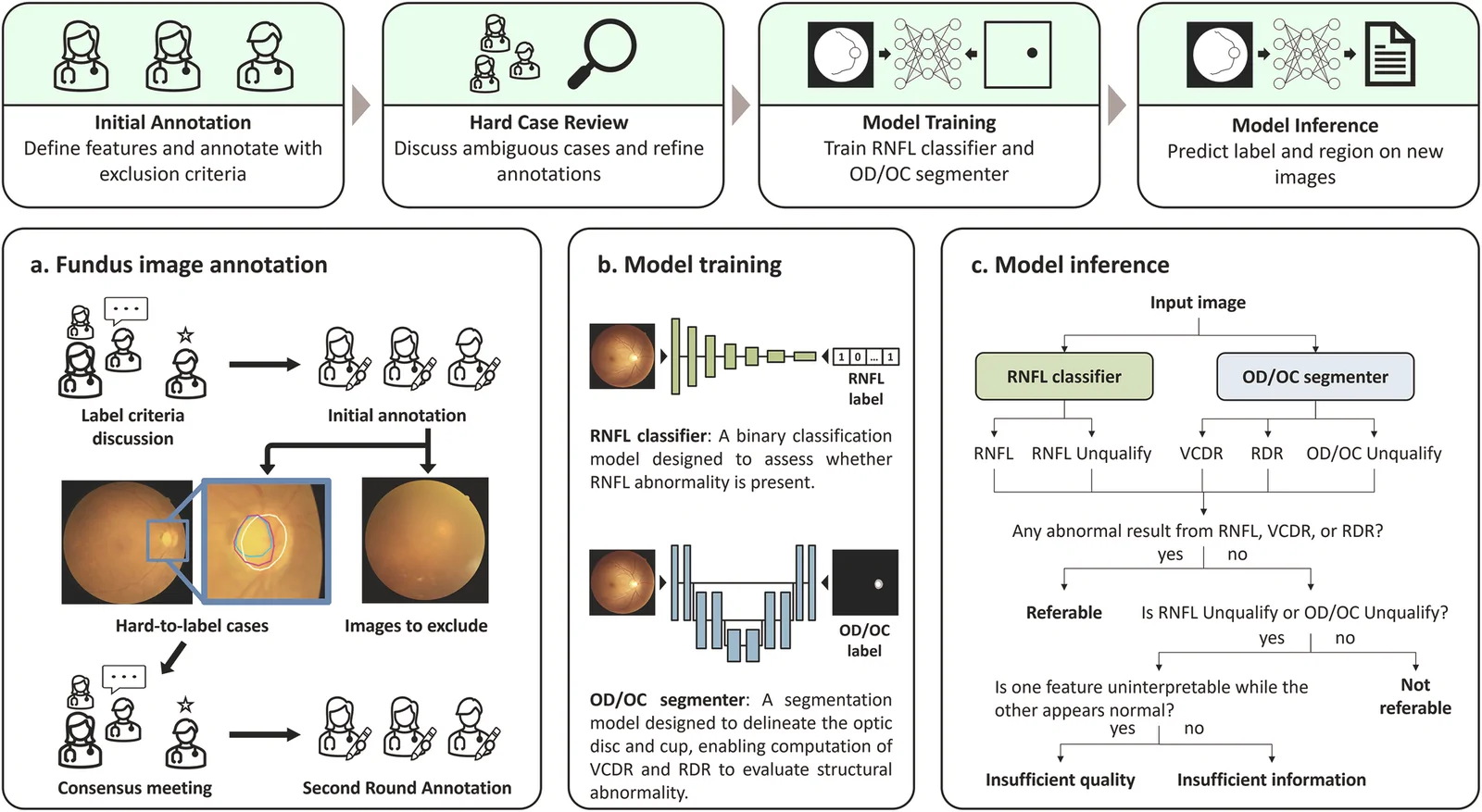
Overall experimental workflow. **a** Fundus image annotation: Each image was independently graded by at least three glaucoma specialists, incorporating initial consensus meetings and subsequent reviews for challenging cases. **b** Model training: This stage included an OD/OC segmenter and an RNFL classifier. **c** Model inference: The final step involved an end-to-end prediction, from fundus image input to a referral decision.

An expert annotation framework was established to ensure consistent labelling across both the development and independent test cohorts. All images were independently reviewed by at least three glaucoma specialists, with final labels assigned by majority vote (Fig. 2a). To maintain consistency, standardised rules were defined through an initial consensus process for RNFL and OD/OC assessments.

For RNFL evaluation, images were classified as normal, abnormal, or uninterpretable. Normal classification required the absence of wedge or diffuse RNFL defects, whereas abnormal status was assigned when wedge or diffuse RNFL defects were present. When assessment of the RNFL was hindered by optic media opacity or high myopia, the result was considered uninterpretable. This label referred to feature-level assessment and was distinct from the earlier exclusion of images from the test cohort.

The assessment of the OD and OC was performed through manual delineation of their boundaries. If the OD and OC margins were indistinct, their positions were inferred from colour variations across multiple filtered views (colour, red-free, green, green + blue and gamma correction) and the course of adjacent blood vessels. When no clear cup was present, a small contour was placed at the centre of the disc to approximate its location. OD/OC images were designated as abnormal if the VCDR was ≥ 0.7 or the RDR was ≤ 0.1. These cut-off values were deliberately established to align with the decision criteria applied during model inference, ensuring methodological consistency between expert-derived ground truth and algorithmic predictions.

Despite applying these standardised rules and conducting the initial consensus process, some challenging cases exhibited residual inter-expert variability, highlighting the necessity for further refinement of the annotation procedure. A second consensus meeting was therefore convened to standardise the annotation workflow for difficult images. Unlike the initial consensus stage, which focused on defining clinical interpretation criteria based on fundus findings, the second meeting focused on how annotations should be performed in practice, particularly for challenging image conditions. For RNFL, the discussion emphasised the consistent use of multiple filtered views when visibility was uncertain, ensuring that reduced contrast or imaging artefacts were not mistaken for true anatomical features. For OD and OC annotation, the discussion centred on applying a unified delineation procedure to ensure sufficiently detailed and stable contour definition when boundaries were ambiguous. Notably, this process did not involve re-labelling previously annotated images. The refined annotation protocol was subsequently applied to the validation subset within the development cohort and to the independent test cohort, thereby forming a two-stage consensus framework designed to improve annotation consistency.

### Model architecture, training and implementation

The system consisted of two core models (Fig. 2b). The first, an RNFL classifier, employed an EfficientNet-B4 backbone [27] with an input size of 288 × 288. It was configured for binary output, using a fixed threshold determined during validation. The second model, an OD/OC segmenter, was a U-Net with an EfficientNet-B4 encoder [28]. The model generated disc and cup masks, from which the VCDR and RDR were computed. Collectively, these two components formed the inference system evaluated in this study, commercially available as VeriSee-GLC (Acer Medical Inc.).

Both models were trained on the development cohort using ImageNet initialisation. The RNFL classifier was trained with a batch size of 24, using binary cross-entropy loss and the Lion optimiser (learning rate 1 × 10−4 with weight decay). Data augmentations included geometric transforms, colour jitter, blur, compression and domain normalisation. The OD/OC segmenter was trained with a batch size of 8 for 50 epochs on a single NVIDIA GTX 1080 Ti. It used an input size of 288×288 and a loss function that combined Dice, binary cross-entropy and Tversky. All segmentation models were implemented in TensorFlow 2.6.0. Additional hyperparameters are detailed in the Supplementary Materials.

### Inference workflow

Each image in the independent test cohort was processed by both the RNFL classifier and the OD/OC segmenter. The RNFL classifier assigned one of three states: normal, abnormal, or uninterpretable. The OD/OC segmenter generated disc and cup masks, from which VCDR and RDR were computed and similarly assigned each case as normal, abnormal, or uninterpretable.

The decision logic, illustrated in Fig. 2c, followed three sequential steps:

1. Abnormality check: An image was labelled as referable if any of the following criteria were met: RNFL abnormal, VCDR ≥ 0.7, or RDR ≤0.1.
2. Uninterpretability check: If step 1 was negative and one or more components were uninterpretable:

- One component was uninterpretable and the other normal; the image was classified as of insufficient quality
- Both components were uninterpretable and the image was classified as insufficient information

Normal findings: If all components were normal and interpretable, the image was labelled as not referable.

Cases classified as uninterpretable due to insufficient quality or insufficient information were excluded from the analysis and counted as system dropouts. However, in clinical practice, such cases are still recommended for referral.

### Evaluation, statistics and explainability

All evaluations were conducted exclusively on the independent test cohort. A case was classified as GON-positive if it met any of the following criteria: RNFL abnormal, VCDR ≥ 0.7, or RDR ≤ 0.1. For comparative analysis, an additional EfficientNet-B4 baseline model was trained to predict image-level GON status directly from fundus photographs. Performance was assessed at two levels. At the component level, model predictions for individual features were compared with the corresponding expert-annotated ground truths. At the decision level, overall system performance was assessed against the final clinical GON diagnosis.

System performance was measured by sensitivity, specificity, positive predictive value (PPV) and negative predictive value (NPV), all reported with 95% exact binomial confidence intervals. This approach was chosen to align with the system’s design, which emulates clinical decision-making by relying on fixed, clinically defined criteria for OD/OC features (VCDR ≥ 0.7 and RDR ≤ 0.1). Accordingly, the Area Under the ROC Curve (AUC) was not reported, as the system’s outputs represent discrete referral states and a continuous ROC curve would not provide an appropriate or clinically meaningful representation of its performance.

For explainability, occlusion maps for the RNFL classifier were generated by systematically masking local regions of the input fundus image and recording the resulting change in the classifier output. Regions associated with a greater drop in the prediction score were considered more influential in the model’s decision. This perturbation-based analysis provided a visual representation of the image areas most relevant to RNFL classification [26]. The OD/OC segmenter produced segmentation overlays highlighting the disc and cup contours, with the corresponding VCDR and RDR values reported to support visual verification and error analysis.

## Results

### Single-feature performance in GON prediction

Table 1 shows that when evaluated as a single-feature predictor, VCDR and RDR exhibited sensitivities of 0.354 (117 GON-positive cases) and 0.491 (136 positives), respectively. Despite their low sensitivities, both VCDR and RDR achieved high predictive values (PPVs) (0.950 and 0.937, respectively) and specificities, serving as reliable indicators that complement RNFL. When VCDR and RDR were combined as cup-disc features, sensitivity improved to 0.524 while specificity and PPV remained high. The RNFL classifier, used as a single-feature predictor, demonstrated higher sensitivity (0.847), moderate specificity (0.827) and moderate PPV (0.728). Given that the GON labels in this study were based on a combination of multiple structural features, a decline in performance with single-feature predictors was anticipated. However, RNFL exhibited the smallest performance reduction, suggesting that RNFL abnormalities may be more closely associated with disease onset than disc morphology alone. Furthermore, most eyes with abnormal cup–disc parameters also exhibited RNFL changes.

**Table 1:** Expert annotations and feature-based analyses.

Summary of expert annotations in the test set			Subset of test set used for model evaluation				
Referral (%)	276 (35.7%)		Referral (%)	268 (35.8%)	Insufficient quality	6	
No Referral (%)	497 (64.3%)		No Referral (%)	481 (64.2%)	Insufficient Information	18	
Total	773		Total	749	Ai drop rate	3.10%	
**Feature-wise prediction of structural annotations**							
Feature Used	Ground Truth	Sensitivity	Specificity	PPV	NPV		
RNFL	RNFL (220 + /517–)	0.900 (0.860–0.940)	0.805 (0.770–0.839)	0.662 (0.609–0.716)	0.950 (0.929–0.970)		
VCDR	VCDR (117 + /616–)	0.769 (0.693–0.846)	0.987 (0.978–0.996)	0.918 (0.864–0.973)	0.957 (0.942–0.973)		
RDR	RDR (136 + /597–)	0.838 (0.776–0.900)	0.958 (0.942–0.974)	0.820 (0.756–0.884)	0.963 (0.948–0.978)		
VCDR + RDR	VCDR + RDR (156 + /577–)	0.814 (0.753–0.875)	0.962 (0.946–0.977)	0.852 (0.795–0.909)	0.950 (0.933–0.968)		
**Comparative performance of structural features in glaucoma detection**							
Feature Used	Ground Truth	Sensitivity (Sen.)	Specificity (Spec.)	PPV	NPV	p (Sen.)	p (Spec.)
Baseline	GON	0.802 (0.755–0.850)	0.807 (0.771–0.842)	0.698 (0.647–0.749)	0.880 (0.849–0.910)	<0.001	0.49
RNFL	GON	0.847 (0.804–0.891)	0.827 (0.794–0.861)	0.728 (0.678–0.778)	0.909 (0.882–0.936)	<0.001	0.25
VCDR	GON	0.354 (0.297–0.411)	0.990 (0.981–0.999)	0.950 (0.908–0.993)	0.737 (0.703–0.770)	<0.001	<0.001
RDR	GON	0.491 (0.431–0.550)	0.982 (0.970–0.994)	0.937 (0.897–0.977)	0.779 (0.746–0.811)	<0.001	<0.001
VCDR + RDR	GON	0.524 (0.465–0.583)	0.978 (0.965–0.991)	0.928 (0.887–0.969)	0.790 (0.757–0.822)	<0.001	<0.001
ALL	GON	0.903 (0.868–0.938)	0.821 (0.787–0.855)	0.738 (0.690–0.785)	0.938 (0.915–0.961)	-	-
**Contingency table of RNFL and VCDR** + **RDR predictions**							
	VCDR + RDR Positive	VCDR + RDR Negative	Total				
RNFL Positive	130	174	304				
RNFL Negative	17	421	438				
Total	147	595	742				

The first section summarises expert annotations of the independent test cohort and the AI drop rate. The second section presents feature-wise predictions for RNFL, VCDR, RDR and VCDR + RDR compared with their structural ground truth. The third section compares GON detection performance across the baseline model, single-feature models, the VCDR + RDR model and the integrated model (ALL). McNemar’s test was applied only to paired comparisons of sensitivity and specificity between each model and the integrated model. The final section presents the contingency table of binary predictions from the RNFL branch and the VCDR + RDR branch.

### Combined-feature model and comparative analysis

Building on these findings, we evaluated whether integrating RNFL, VCDR and RDR could enhance diagnostic performance for GON. The combined-feature model achieved a sensitivity of 0.903, specificity of 0.821, PPV of 0.738 and NPV of 0.938, offering broader coverage than any single feature. To formally assess these improvements, McNemar’s test was applied to paired predictions from the combined-feature model and each alternative configuration, including a simple binary baseline model trained directly on the final GON labels, RNFL alone, VCDR alone, RDR alone and VCDR + RDR. The combined model showed significantly higher sensitivity than the baseline model (*p* < 0.001), with no significant difference in specificity (*p* = 0.49). A similar pattern was observed when compared with RNFL alone, with significantly improved sensitivity (*p* < 0.001) and comparable specificity (*p* = 0.25). Moreover, the integrated model (ALL) demonstrated markedly higher sensitivity than the VCDR + RDR configuration, indicating that RNFL information identified additional GON cases not captured by disc-cup features alone. This complementary pattern was further supported by the contingency table of RNFL and VCDR + RDR predictions. The association between the two branches was significant (*p* < 0.001), while the discordant predictions indicated that the two branches were related but not identical.

### Annotation consistency after consensus meeting

**Fig. 3:**
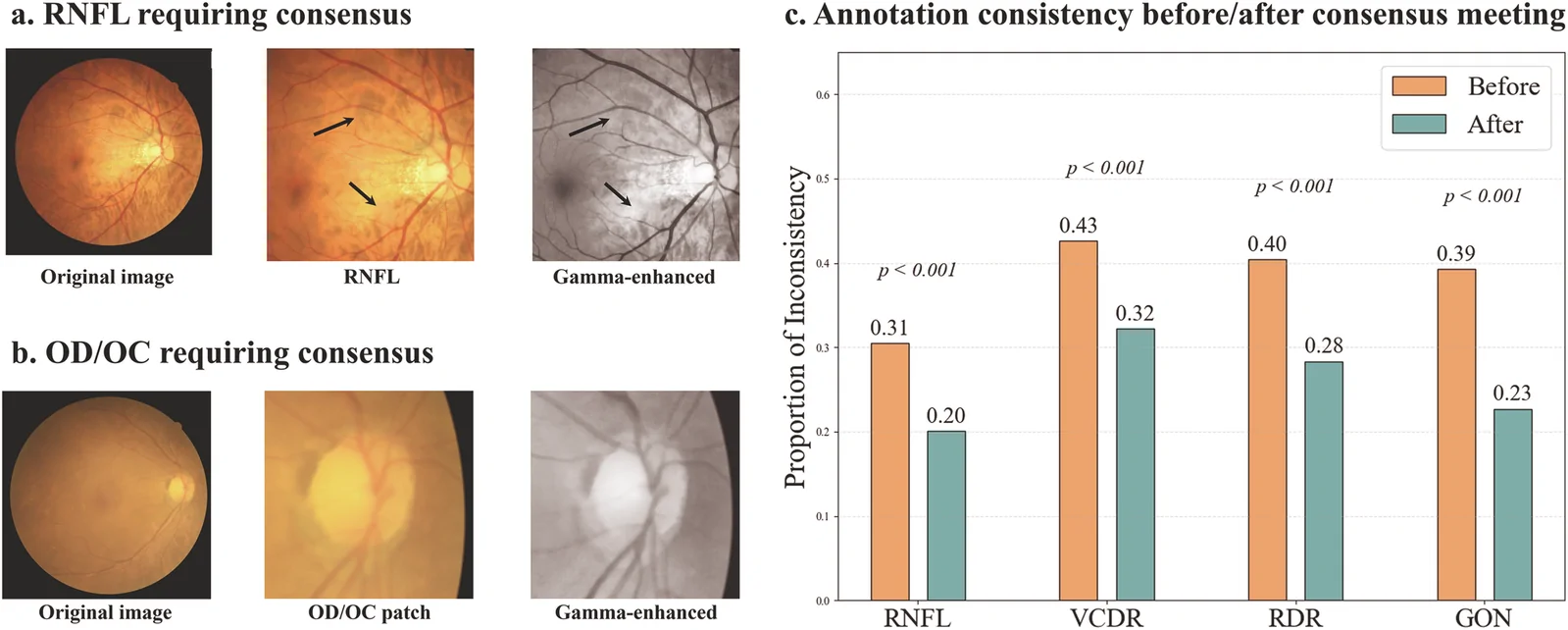
Annotation process for challenging cases. **a** A case of high myopia with unclear RNFL visibility, clarified by gamma correction. **b** A cataract case with indistinct disc boundaries, delineated using vessel guidance after image correction. **c** Illustration of improved annotation consistency following a consensus meeting.

Following the consensus procedure, disagreement rates decreased across all four target labels (Fig. 3c). Specifically, the disagreement rates for GON fell from 39% to 23%, for VCDR from 43% to 32%, for RDR from 40% to 28% and for RNFL from 31% to 20%. These reductions were significant for all four labels (two-sided proportion z-test, all *p* < 0.001). Some images remained ambiguous even after the consensus meeting and final labels were therefore assigned by majority vote among glaucoma specialists. All validation data from the development cohort and the independent test cohort were annotated according to the refined consensus workflow, whereas the training set included annotations from both before and after the consensus meeting.

## Discussion

To ensure a rigorous evaluation of model reliability, we examined both the consistency of human annotations and the interpretability of model predictions. This section is structured into two parts. First, we investigated the impact of consensus annotation on inter-clinician label agreement. Subsequently, we explored complementary diagnostic behaviour exhibited by RNFL and OD/OC-based models through representative case analyses.

### Impact of consensus meetings on annotation reliability

Despite prior agreement on annotation criteria, the initial annotations revealed noticeable inter-expert disagreement. This reflects the challenge of delineating anatomical variations. Figure 3 presents two examples illustrating sources of annotation. In Fig. 3a, an image of a highly myopic eye exhibited insufficient contrast in the retinal pigment epithelium, rendering RNFL evaluation inconclusive in standard colour mode. Application of gamma correction to the fundus image revealed diffuse loss in the superior and inferior temporal regions. Figure 3b illustrates a case involving a cataract, which obscured the cup contour in colour mode. The interpreting physician reviewed the gamma-corrected image and delineated the cup contour based on colour contrast and the angulation of vessels on the OD. These examples highlight the necessity of a standardised annotation workflow after initial agreement on grading criteria.

### Model complementarity and case visualisation

**Fig. 4:**
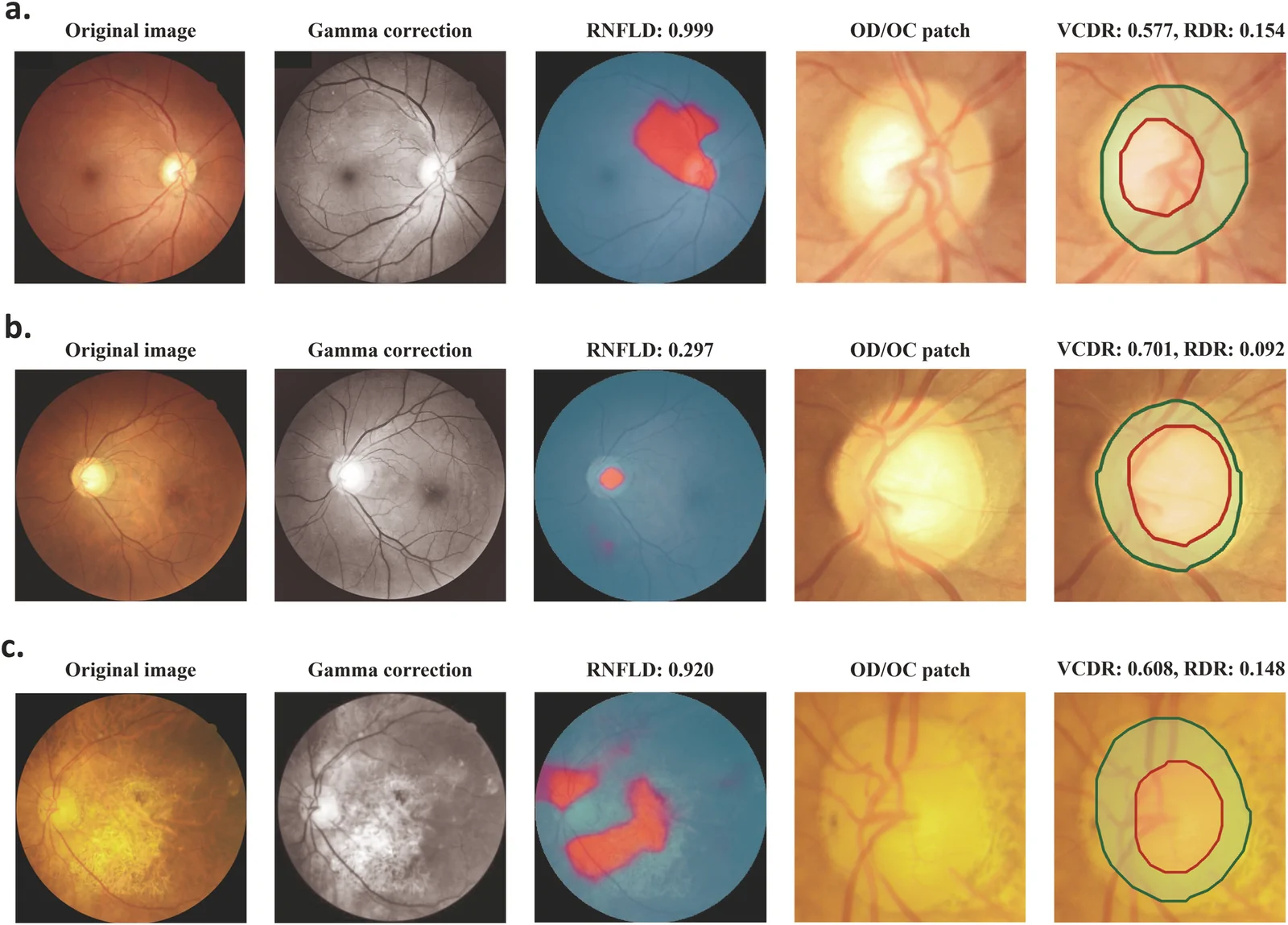
Complementary approach to AI-based glaucoma diagnosis. **a** RNFL abnormality detected while VCDR and RDR remain within normal limits. **b** Abnormal VCDR and RDR are observed despite a normal RNFL; in both (**a**) and **b** assessments by glaucoma assessments align with the AI interpretation. **c** AI detects abnormal RNFL with normal VCDR and RDR, whereas specialists confirm normal VCDR and RDR but find the RNFL uninterpretable.

The RNFL classifier and OD/OC segmenter provided distinct diagnostic insights. In some cases, the RNFL was classified as abnormal, while the OD/OC appeared normal. In other instances, the disc–cup structure was abnormal despite a normal RNFL. These findings underscore the limitations of relying on a single structural feature, which may result in missed diagnoses. Furthermore, a subset of images was deemed insufficient by human experts, reflecting cases in which structural assessment was inherently challenging. To illustrate both complementary diagnostic patterns and these challenging cases, three representative cases were selected in which ground-truth annotations demonstrated complementary patterns or insufficient image quality: one labelled RNFL abnormal with normal OD/OC, one labelled RNFL normal with abnormal OD/OC and one graded as insufficient by human experts. The full analysis workflow is visualised in Fig. 4, with each panel depicting a five-step visual workflow: the original fundus image, the gamma-corrected image, the RNFL prediction, the cropped OD and the OD/OC prediction, along with the corresponding RDR and VCDR values.

In the first case (Fig. 4a), ground truth annotations indicated a normal VCDR and RDR but an abnormal RNFL. A superior temporal wedge-shaped RNFL defect was evident in the original fundus image and became more apparent following gamma correction. The RNFL classifier detected this defect with high confidence (prediction = 0.999) and the occlusion heat map was accurately localised to the affected area. The OD/OC segmenter yielded VCDR and RDR values of 0.577 and 0.154, respectively, both within normal limits. In the second case (Fig. 4b), the ground truth annotations revealed an abnormal VCDR and RDR, but a normal RNFL. The RNFL was confirmed to be normal in the gamma-corrected image. Accordingly, our model classified the RNFL as normal with a low prediction score (0.297). In contrast, the segmentation model reported VCDR and RDR measurements of 0.701 and 0.092, respectively, indicating a clear abnormality in the disc-cup structure. In the third case (Fig. 4c), the fundus photograph was graded as uninterpretable for RNFL status by human experts. The presence of a tessellated fundus made it particularly challenging to evaluate the RNFL; while diffuse atrophy might be suspected, a definitive clinical assessment was precluded. Consequently, the model identified the VCDR and RDR as normal and detected an abnormal RNFL status. This still resulted in a referral decision and illustrates that low-quality or ambiguous images remain a significant hurdle for both automated analysis and human grading. This example serves as a critical comparison point and demonstrates that such diagnostic difficulties are not specific to the AI model but are equally reflected in expert judgment.

Collectively, these findings underscore the significance of RNFL abnormalities as reliable indicators of GON in fundus photographs. The results demonstrate that such changes can be effectively recognised by deep learning models. Reliance solely on OD/OC analysis may reduce sensitivity, particularly when cup–disc ratios appear normal despite defects. When incorporated as a complementary feature, OD/OC segmentation enhances overall GON recognition and aligns with the multi-feature assessment strategies commonly employed in clinical glaucoma diagnosis.

### Clinical scope and applicability

The VeriSee-GLC was developed as a fundus-based screening tool to emulate the clinical reasoning of glaucoma specialists by incorporating structural interpretations of the OD and RNFL. This design is supported by recent evidence suggesting that combining multiple structural markers can improve the performance of fundus-based glaucoma screening. For instance, Sharma et al. demonstrated that sensitivity improved from 0.5652 to 0.8053 when multiple glaucomatous signs were integrated, rather than relying on a single binary model [24]. Our case analyses similarly support the complementary diagnostic value of RNFL and OD/OC cues, particularly when a single feature may not exhibit clear abnormalities. Accordingly, VeriSee-GLC is intended to support early identification of suspicious cases using widely accessible fundus imaging.

In routine glaucoma care, diagnosis also relies on additional clinical information, such as OCT, visual field testing and other ophthalmic examinations. The role of VeriSee-GLC is not to replace comprehensive diagnosis but rather to assist in primary screening and referral decisions in fundus-based settings. In our proposed clinical workflow, the presence of an RNFL defect serves as a direct trigger for referral, regardless of VCDR or RDR values. In the absence of RNFL defects, eyes with enlarged VCDR or thinned RDR are advised to undergo regular follow-up photography rather than immediate advanced testing. This tiered strategy facilitates more efficient case prioritisation and helps alleviate unnecessary clinical burden.

The interpretation of screening performance should also account for the cohort composition. The independent test cohort was hospital-based, with a relatively high GON prevalence of 35.8%. This characteristic should be considered when interpreting the observed PPV, particularly in broader community screening settings with lower disease prevalence. Furthermore, differences in case composition should also be taken into account when interpreting sensitivity and specificity across screening populations. Nevertheless, the current results support the potential utility of the system as a practical screening aid in settings where fundus photography is used for early case identification.

To improve diagnostic consistency and reduce structural confounding, eyes with myopia greater than –10D, tigroid fundus changes and poor RNFL visibility were excluded. These criteria define the parameters within which the current results should be interpreted. In cases where RNFL assessment is challenging, the screening protocol was adapted to prioritise VCDR and RDR as the primary indicators, with a referral recommended if either parameter is abnormal. Within this clinical scope, the proposed framework provides a meaningful and flexible strategy for identifying eyes that warrant further glaucoma evaluation.

## Summary

### What is known about this topic

- Most current AI models for glaucoma detection rely on a single feature, predominantly the OD/OC assessment.
- Clinical diagnosis of glaucoma is typically based on a combination of signs, rather than OD/OC evaluation alone.
- Inter-observer variability frequently arises among specialists during image annotation, even after an initial consensus meeting and such discrepancies are rarely resolved.

### What this study adds

- Our deep learning model directly detects RNFL abnormalities from fundus images, demonstrating that these signs can be computationally captured.
- The integration of RNFL predictions with OD/OC assessment provides complementary diagnostic information, thereby improving sensitivity in detecting glaucomatous signs.
- Conducting a second consensus meeting during annotation enhances inter-rater agreement and yields a more reliable dataset for model training.

## Supplementary information

Supplementary Materials

## Data Availability

The datasets generated and analysed during the current study are not publicly available due to participant confidentiality and institutional review board restrictions.
