# Supplementary material for: Clinically aligned artificial intelligence for glaucoma diagnosis: enhancing retinal nerve fibre layer interpretation from fundus images
Source: Eye (Lond). 2026 Jul 23;40(15):2424–31. doi: 10.1038/s41433-026-04758-w (PMC13675183; doi:10.1038/s41433-026-04758-w)
Supplement: Supplementary file 1 [file 41433_2026_4758_MOESM1_ESM.docx]

**Supplementary Materials**

Clinically Aligned Artificial Intelligence for Glaucoma Diagnosis: Enhancing Retinal Nerve Fibre Layer Interpretation from Fundus Images

# Supplementary Methods

This supplementary file summarises the principal training settings used for the retinal nerve fibre layer (RNFL) classifier, the optic disc/optic cup (OD/OC) segmenter, and the image-level baseline glaucomatous optic neuropathy (GON) classifier. The settings are reported to complement the model development description in the main manuscript and are limited to parameters relevant for reproducibility and interpretation.

## Supplementary Table 1. Principal training settings for the RNFL classifier.

| **Item** | **Setting** |
| --- | --- |
| Model task | Binary classification of RNFL abnormality from fundus photographs |
| Model role in the study | Primary lesion-level branch for detecting visible RNFL defects on fundus photography |
| Architecture or backbone | EfficientNet-B4 |
| Input | Colour fundus photograph |
| Input size | 288 x 288 pixels |
| Initialisation | ImageNet pretraining |
| Output | Single sigmoid output |
| Training label or target | Expert-derived RNFL status after the consensus annotation workflow |
| Positive or abnormal definition | Presence of wedge or diffuse RNFL defects; uninterpretable cases were handled according to the inference workflow in the main manuscript |
| Loss function | Binary cross-entropy |
| Optimiser | Lion |
| Learning rate | 1 x 10^-4 |
| Batch size | 24 |
| Training epochs | 50 |
| Threshold or decision rule | Fixed operating threshold determined using the validation set |
| Data augmentation | Geometric transformation, colour jitter, blur, image compression |
| Implementation notes | Configured as the RNFL branch of the clinically aligned framework |

## Supplementary Table 2. Principal training settings for the OD/OC segmenter.

| **Item** | **Setting** |
| --- | --- |
| Model task | Segmentation of the optic disc and optic cup |
| Model role in the study | Structural branch used to derive VCDR and RDR for OD/OC assessment |
| Architecture or backbone | U-Net with an EfficientNet-B4 encoder |
| Input | Colour fundus photograph |
| Input size | 288 x 288 pixels |
| Initialisation | ImageNet pretraining for the encoder |
| Output | Optic disc and optic cup masks |
| Training label or target | Expert delineations of the optic disc and optic cup |
| Positive or abnormal definition | Abnormal OD/OC status was defined as VCDR >= 0.7 or RDR <= 0.1 during inference |
| Loss function | Combined Dice loss, binary cross-entropy, and Tversky loss |
| Optimiser | Model-specific optimiser configuration used during segmentation training |
| Learning rate | 1 x 10^-4 |
| Batch size | 8 |
| Training epochs | 50 |
| Threshold or decision rule | VCDR >= 0.7 or RDR <= 0.1 was considered abnormal |
| Data augmentation | Geometric transformation, colour jitter, blur, image compression |
| Implementation notes | Implemented as an EfficientUnet configuration with Mish activation and layer normalisation |

## Supplementary Table 3. Principal settings for the image-level baseline GON classifier.

| **Item** | **Setting** |
| --- | --- |
| Model task | Direct image-level classification of GON status from fundus photographs |
| Model role in the study | Comparative baseline for assessing the value of explicitly modelling RNFL and OD/OC features |
| Architecture or backbone | EfficientNet-B4 |
| Input | Colour fundus photograph |
| Input size | 288 x 288 pixels |
| Initialisation | ImageNet pretraining |
| Output | Single sigmoid output |
| Training label or target | Image-level GON status |
| Positive or abnormal definition | An image was considered GON-positive if it showed RNFL abnormality, VCDR ≥ 0.7, or RDR ≤ 0.1. It was considered GON-negative only when RNFL abnormality was absent and both VCDR and RDR remained within the normal range. |
| Loss function | Binary cross-entropy |
| Optimiser | Lion |
| Learning rate | 1 x 10^-4 |
| Batch size | 24 |
| Training epochs | 50 |
| Threshold or decision rule | Fixed operating threshold determined using the validation set |
| Data augmentation | Geometric transformation, colour jitter, blur, image compression |
| Implementation notes | The baseline was implemented as a direct image-level GON classifier, independent of the proposed framework’s RNFL and OD/OC decision branches. |
